# Supplementary material for: Mapping the adaptive landscape of Batesian mimicry using 3D-printed stimuli
Source: Nature. 2025 Jul 2;644(8077):706–13. doi: 10.1038/s41586-025-09216-3 (PMC12367557; doi:10.1038/s41586-025-09216-3)
Supplement: Supplementary file 1 — Reporting Summary [file 41586_2025_9216_MOESM1_ESM.pdf]

nature portfolio

Corresponding author(s): Christopher Taylor

Last updated by author(s): 15/05/2025

Reporting Summary

Nature Portfolio wishes to improve the reproducibility of the work that we publish. This form provides structure for consistency and transparency in reporting. For further information on Nature Portfolio policies, see our Editorial Policies and the Editorial Policy Checklist.

Statistics

For all statistical analyses, confirm that the following items are present in the figure legend, table legend, main text, or Methods section.

n/a	Confirmed	
☐	☒	The exact sample size (*n*) for each experimental group/condition, given as a discrete number and unit of measurement
☐	☒	A statement on whether measurements were taken from distinct samples or whether the same sample was measured repeatedly
☐	☒	The statistical test(s) used AND whether they are one- or two-sided<br>*Only common tests should be described solely by name; describe more complex techniques in the Methods section.*
☐	☒	A description of all covariates tested
☐	☒	A description of any assumptions or corrections, such as tests of normality and adjustment for multiple comparisons
☐	☒	A full description of the statistical parameters including central tendency (e.g. means) or other basic estimates (e.g. regression coefficient) AND variation (e.g. standard deviation) or associated estimates of uncertainty (e.g. confidence intervals)
☐	☒	For null hypothesis testing, the test statistic (e.g. *F*, *t*, *r*) with confidence intervals, effect sizes, degrees of freedom and *P* value noted<br>*Give P values as exact values whenever suitable.*
☒	☐	For Bayesian analysis, information on the choice of priors and Markov chain Monte Carlo settings
☒	☐	For hierarchical and complex designs, identification of the appropriate level for tests and full reporting of outcomes
☒	☐	Estimates of effect sizes (e.g. Cohen's *d*, Pearson's *r*), indicating how they were calculated

Our web collection on statistics for biologists contains articles on many of the points above.

Software and code

Policy information about availability of computer code

Data collection	3DSOM Pro v5<br>Deformetrica 4.3.0<br>Blender 2.9
Data analysis	BORIS 8.19.3<br>R v4.3.0 with packages tidyverse 2.0.0, lme4 1.1, nlme 3.1, emmeans 1.10, and MuMIn 1.47

For manuscripts utilizing custom algorithms or software that are central to the research but not yet described in published literature, software must be made available to editors and reviewers. We strongly encourage code deposition in a community repository (e.g. GitHub). See the Nature Portfolio guidelines for submitting code & software for further information.

Data

Policy information about availability of data

All manuscripts must include a data availability statement. This statement should provide the following information, where applicable:

- Accession codes, unique identifiers, or web links for publicly available datasets
- A description of any restrictions on data availability
- For clinical datasets or third party data, please ensure that the statement adheres to our policy

Data have been deposited at the NERC Environmental Information Data Centre. Some datasets are currently under embargo until April/May 2025 which can be

lifted if accepted for publication at an earlier date.
Stimuli: "Scanned 3D images and 3D printable images based on combinations of features of Diptera and Hymenoptera collected from the UK in 2021-22"
https://doi.org/10.5285/05169766-7355-4c3c-8ade-091db0583f9d
Wild bird experiments: "Great tit behavioural responses to 3D-printed insect replicas, featuring combinations of traits from wasps and flies, in Madingley Wood, Cambridge, UK, 2021-2023" (under embargo until 1 April 2025)
https://doi.org/10.5285/a1c9b0cc-5585-49c5-a38f-fe05240edccf
Trait Salience: "Chick behavioural responses to 3D-printed insect replicas, featuring combinations of traits from wasps and flies" (under embargo until 1 May 2025)
https://doi.org/10.5285/45391184-603e-4284-bb3c-9c8c6bf856ab
Invertebrate Predators: "Invertebrate behavioural responses to 3D-printed insect replicas, featuring combinations of traits from wasps and flies, in laboratory trials" (under embargo until 1 April 2025)
https://doi.org/10.5285/ee7ba05a-449b-466e-840c-8de1d3f1d4d1

Research involving human participants, their data, or biological material

Policy information about studies with human participants or human data. See also policy information about sex, gender (identity/presentation), and sexual orientation and race, ethnicity and racism.

Reporting on sex and gender	No human participants/data
Reporting on race, ethnicity, or other socially relevant groupings	No human participants/data
Population characteristics	No human participants/data
Recruitment	No human participants/data
Ethics oversight	No human participants/data

Note that full information on the approval of the study protocol must also be provided in the manuscript.

Field-specific reporting

Please select the one below that is the best fit for your research. If you are not sure, read the appropriate sections before making your selection.

☐ Life sciences ☐ Behavioural & social sciences ☒ Ecological, evolutionary & environmental sciences

For a reference copy of the document with all sections, see nature.com/documents/nr-reporting-summary-flat.pdf

Ecological, evolutionary & environmental sciences study design

All studies must disclose on these points even when the disclosure is negative.

Study description

All experiments tested the responses of predators towards 3D-printed stimuli of varying levels of mimetic accuracy, associated with different levels of reward or punishment.
Discrimination Ability: recorded the order in which wild great tits opened dishes bearing 3D stimuli to obtain potential mealworm reward. Three replicate feeding stations with 49 dishes on each, reset and repeated in daily sessions for three weeks. Predictors were 3D stimulus type, presence of mealworm, position of dish (edge vs centre).
Multiple Models experiment: recorded the order in which wild great tits opened dishes bearing 3D stimuli to obtain potential mealworm reward. Six replicate feeding stations divided between two treatments: "one model" or "two model" based on the number of model (unrewarding) stimulus types used. 49 dishes at each feeder, reset daily over the course of six weeks. Predictors were treatment, 3D stimulus type, presence of mealworm, position of dish (edge vs centre).
Validation test: Recorded the order in which wild great tits opened dishes bearing 3D stimuli to obtain potential mealworm reward. Four replicate feeding stations with 24 dishes on each, reset and repeated in daily sessions for five days. Predictors were 3D stimulus type, presence of mealworm, position of dish (edge vs centre).
Trait Salience experiment: recorded the time taken for chicks to approach and peck at dishes bearing 3D stimuli and (for some stimuli) containing a mealworm. 1445 presentations to 30 chicks. Predictors were chick id, day within experimental run (1-4), batch (two different batches of chicks tested on different dates), whether or not it was the first presentation of a trial, and 3D stimulus type.
Invertebrate Predators experiment: recorded behavioural responses of praying mantises (n= 40 presentations, 8 individuals), jumping spiders (n = 57 presentations, 9 individuals) and crab spiders (n = 50 presentations, 50 individuals) towards 3D printed stimuli. Mantises and jumping spiders received all different types of 3D stimulus, whereas crab spiders were randomly assigned to a single stimulus type. Predictors were stimulus type and individual id.

Research sample

Discrimination Ability, Multiple Models experiments and Validation test: a population of great tits (Parus major) within Madingley Wood, Cambridge, UK, some PIT tagged (estimated 51-71%). Birds that visited our feeding stations were all adult plumage, but sex and exact age were not recorded. This population was chosen as consisting of wild generalist insectivorous predators expected to show propensity and ability to learn to obtain food from our experimental set up.
Trait Salience: domestic chicks (Gallus gallus domesticus) obtained from a commercial hatchery on the day after hatching, with testing phase of the experiment when they were 17-20 days old. At this age they were not identifiable to sex. They were chosen as

	generalist insectivorous predators that could be housed in a laboratory from hatching and therefore of known prior experience regarding food types. Invertebrate Predators: Praying mantises of three species (Rhombodera kirbyi (n = 5), Polyspilota aeruginosa (n = 1) and Pseudoxyops perpulchra (n = 2)), jumping spiders (Phidippus audax) and crab spiders (Synema globosum). Crab spiders included both male and female, others were not sexed. These subjects were chosen to represent a range of visual types and predation strategies from invertebrates that are known or suspected to prey on Diptera and Hymenoptera.
Sampling strategy	Discrimination Ability and Multiple Models experiments, Validation test: sample sizes were constrained mainly by the birds' seasonal behaviour and the number of birds present in the population that chose to visit our feeding stations.<br>Trait Salience and Invertebrate Predators experiments: sample sizes were calculated to give repeats of around n=10 for each stimulus type, minimising the numbers of laboratory animals required.
Data collection	Discrimination Ability and Multiple Models experiments, Validation test: video recordings of bird behaviour were taken using a motion-sensitive trail camera and combined with manual records taken of which dishes were open at the end of a given session. Data were recorded by CHT, DB, SB, AC, KC, JRD, SRG, EO, ALP and TR.<br>Trait Salience experiment: behavioural data were noted by the experimenters (CHT and DJGW) at the time of the experiment, and videos were recorded which were later analysed by CHT and DB to determine latency to attack the dishes.<br>Invertebrate Predators: instances of particular behaviours were noted by the experimenters during trials and in some cases verified using video recordings. Data were recorded by TD and RL (jumping spiders), HR (praying mantises), JD, HJJ, JR, JSa (crab spiders).
Timing and spatial scale	Discrimination Ability experiment: experiment ran from December 2021 to May 2022, with the testing phase beginning on 5th April 2022. Feeders were spaced within an area approximately 150 m x 150 m.<br>Multiple Models experiment: ran from October 2022 to March 2023, with the testing phase beginning on 13th January 2023.<br>Validation test: ran from October to December 2023, with the testing phase beginning on 18th December.
Data exclusions	Discrimination Ability experiment: an additional two feeders were removed from the experiment before the start of the testing phase as they did not receive sufficient great tit visits.<br>Multiple Models experiment: some dishes were opened by mice, which were excluded from the analysis.<br>Validation test: no exclusions<br>Trait Salience experiment: we drew a predetermined cut-off of 13 out of 16 successes during a single trial in the training phase. Chicks that did not reach this cut-off did not proceed to the testing phase. We also excluded testing trials where there was <0.1s in latency to attack towards the fly and wasp stimuli, on the basis that chicks were not showing detectable discrimination among stimulus types.<br>Invertebrate Predators experiment: no exclusions.
Reproducibility	No extra tests of reproducibility were carried out beyond the repetition inherent in the experimental design described above.
Randomization	Discrimination Ability and Multiple Models experiments, Validation test: within each feeder, a defined set of stimuli were allocated to dishes at random. Treatments for the Multiple Models experiment were assigned to feeders according to spatial grouping, to minimise the numbers of birds that would visit both treatment types.<br>Trait Salience: each trial included presentations of 6 fly and 6 wasp stimuli, as well as 4 probe types that were selected at random from a defined pool (without replacement, to ensure roughly equal sample sizes for each type). Within a trial, order was randomised with the constraint that each half (8 presentations) would contain 3 fly, 3 wasp and 2 probe presentations, to prevent the chicks experiencing long runs of the same stimulus type or reward status.<br>Invertebrate Predators: for mantises and jumping spiders, order of trials was randomised within the testing phase. For crab spiders, individuals were assigned to treatments at random.
Blinding	Data transcribed from video recordings were carried out with reference to dish location or trial number and without knowledge of the stimulus type in question (video resolution was not sufficient to reliably identify stimuli visually).

Did the study involve field work? ☒ Yes ☐ No

Field work, collection and transport

Field conditions	Highly variable. During one period of the Multiple Models experiment data collection was paused as snow and below-zero temperatures were causing the dishes to freeze shut. Detailed weather records were not maintained due to the long period of data collection.
Location	Madingley Wood, Cambridgeshire, UK (52.217 N, 0.049 E)
Access & import/export	Permission for carrying out fieldwork was obtained from University Farm & Rural Estate (University of Cambridge) who manage the woodland
Disturbance	Disturbance from researcher visits was minimised as most data recording was done by trail cameras and each feeder was visited only once per day, with visits taking about half an hour per feeder.

Reporting for specific materials, systems and methods

We require information from authors about some types of materials, experimental systems and methods used in many studies. Here, indicate whether each material, system or method listed is relevant to your study. If you are not sure if a list item applies to your research, read the appropriate section before selecting a response.

Materials & experimental systems

n/a	Involved in the study
x	Antibodies
x	Eukaryotic cell lines
x	Palaeontology and archaeology
	x Animals and other organisms
x	Clinical data
x	Dual use research of concern
x	Plants

Methods

n/a	Involved in the study
x	ChIP-seq
x	Flow cytometry
x	MRI-based neuroimaging

Animals and other research organisms

Policy information about studies involving animals; ARRIVE guidelines recommended for reporting animal research, and Sex and Gender in Research

Laboratory animals	Domestic chicks (Gallus gallus domesticus) were obtained from a commercial hatchery on the day after hatching, with testing phase of the experiment when they were 17-20 days old. Praying mantises of three species (Rhombodera kirbyi (n = 5), Polyspilota aeruginosa (n = 1) and Pseudoxyops perpulchra (n = 2)) and jumping spiders (Phidippus audax) were obtained from commercial sellers at a range of ages from 3rd instar to adult.
Wild animals	Wild birds were free to come and go from feeding stations at will, they were not captured at any point during the experiment. Crab spiders (adult, exact age unknown) were captured from the wild by hand in specimen tubes around the Quinta de São Pedro field research station (38.568 N, 9.193 W) and surrounding areas of Sobreda, Portugal, and transported on foot. They were kept for approximately 48 h before being released in the same location.
Reporting on sex	Most of our study organisms were not sexable at the time of experiment. Those where sex was identifiable consisted of a mix of sexes, and we have no reason to assume sex biases in the other cases. We did not analyse effects of sex as this was not a variable of interest and would only have been possible in limited sub-sets of the data.
Field-collected samples	No laboratory samples collected from the field
Ethics oversight	The Trait Salience experiment was approved by Newcastle University AWERB committee (project ID 966). Wild bird experiments (Discrimination Ability and Multiple Models) were approved by AWERB committees at University of Nottingham (project ID 260) and University of Cambridge (ref. NR2022/60).

Note that full information on the approval of the study protocol must also be provided in the manuscript.

Plants

Seed stocks	*Report on the source of all seed stocks or other plant material used. If applicable, state the seed stock centre and catalogue number. If plant specimens were collected from the field, describe the collection location, date and sampling procedures.*
Novel plant genotypes	*Describe the methods by which all novel plant genotypes were produced. This includes those generated by transgenic approaches, gene editing, chemical/radiation-based mutagenesis and hybridization. For transgenic lines, describe the transformation method, the number of independent lines analyzed and the generation upon which experiments were performed. For gene-edited lines, describe the editor used, the endogenous sequence targeted for editing, the targeting guide RNA sequence (if applicable) and how the editor was applied.*
Authentication	*Describe any authentication procedures for each seed stock used or novel genotype generated. Describe any experiments used to assess the effect of a mutation and, where applicable, how potential secondary effects (e.g. second site T-DNA insertions, mosiacism, off-target gene editing) were examined.*
